# Supplementary material for: Thiol-Mediated Uptake of a Cysteine-Containing Nanobody for Anticancer Drug Delivery
Source: ACS Cent Sci. 2023 May 11;9(6):1111–8. doi: 10.1021/acscentsci.3c00177 (PMC10311659; doi:10.1021/acscentsci.3c00177)
Supplement: Supplementary file 1 — oc3c00177_si_001.pdf [file oc3c00177_si_001.pdf]

Supplementary Information

Thiol-mediated Uptake of a Cysteine-containing Nanobody for Anti-Cancer Drug Delivery

Felix Goerdeler[1,2], Emelie E. Reuber[1,2,§], Jost Lühle[1,2,§], Anika Freitag[1,3], Sabrina Leichnitz[1,2], Ruslan Nedielkov[3], Raluca Groza[2], Helge Ewers[2], Heiko M. Möller[3], Peter H. Seeberger[1,2,*] and Oren Moscovitz[1,*]

[1]Department of Biomolecular Systems, Max Planck Institute of Colloids and Interfaces, 14476 Potsdam, Germany

[2]Institute of Chemistry and Biochemistry, Freie Universität Berlin, 14195 Berlin, Germany

[3]Institute of Chemistry, University of Potsdam, 14476 Potsdam, Germany

[§]These authors contributed equally to this work.

[*]correspondence should be addressed to peter.seeberger@mpikg.mpg.de or oren.moscovitz@mpikg.mpg.de

Experimental Section

1. Nanobody expression and purification
2. Site-directed mutagenesis of CB2
3. NMR measurements of CB2 and ^{C105S}CB2
4. Cell culture
5. Nanobody binding assays and determination of apparent affinity
6. Thiol-binding inhibition assays
7. Determination of surface thiol levels
8. Synthesis of Rha_3
9. Rha_3 conjugation to CB2
10. Glycan array
11. Complement activation assay
12. Cy3 conjugation to CB2
13. Endocytosis inhibition assay
14. Live-cell intracellular traffic assay
15. Colocalization analysis pipeline
16. MMAE conjugation to CB2
17. Drug delivery assay

List of Supplementary Figures

1. CB2 binding to SC-1 cells (apparent affinity/time course) and additional cell lines
2. CB2 dimerization/mutant and NMR spectra
3. DTT resilience of aCD19-Ab and CB2 binding with TCEP/glutathione
4. Cell surface staining with Alexa647-maleimide
5. NMR spectra of Rha_3
6. Rha_3 conjugation to CB2
7. Cy3 conjugation to CB2
8. Endocytosis inhibition assay with nanobody-Cy3 conjugates
9. MMAE conjugation to CB2

Experimental Section

No unexpected or unusually high safety hazards were encountered.

Nanobody expression and purification

The sequence of CB2 was synthesized by Synbio Technologies (New Jersey, USA) with a C-terminal glycine-serine linker ($[G_4S]_3$) followed by a sortag (LPETG) and a His_6 tag in a pET-28b(+) vector. The plasmid was transformed into *E. coli* SHuffle cells (NEB, Ipswich, MA, USA) and the protein was expressed in TB medium via induction with 0.5 mM IPTG at 16°C overnight. Bacteria were lysed using a French press and CB2 was purified using gravity nickel-NTA chromatography followed by size exclusion chromatography on an ÄKTApurifier with a Superdex 75 16/600 column (GE Healthcare). All steps of nanobody purification were validated by SDS-PAGE. CB2 was purified in the presence of 0.75 mM dithiothreitol to prevent dimerization.

Site-directed mutagenesis of CB2

The ^{C105S}CB2 mutant was generated in-lab via overlap extension PCR as described elsewhere[1]. Briefly, forward and reverse primers containing the mutation were ordered from IDT (Coralville, IA, USA) and two separate PCRs were performed using each primer and the corresponding T7 standard primer. In a second PCR, both fragments were joined via overlap extension and the final product was cloned into the pET-28b(+) backbone. Expression of the mutant was carried out as described before.

NMR measurements of CB2 and ^{C105S}CB2

For NMR measurements, CB2 and ^{C105S}CB2 were expressed in labeled M9 minimal medium containing 1 g/L ^{15}N-NH_4Cl (Merck), 1 g/L NaCl, 3 g/L KH_2PO_4, 6 g/L Na_2HPO_4, pH 7.4, 2 mM $MgSO_4$, 100 µM $CaCl_2$, vitamin mix and 100 mg/L ^{15}N-Celtone (Cambridge Isotope Laboratories) via induction with 0.5 mM IPTG at 16°C, overnight. The bacteria were lysed, and proteins purified as described above. After purification, the proteins were concentrated using Amicon-4 Ultra (MWCO = 3 kDa) and transferred to PBS buffer supplemented with 0.75 mM DTT, 8 mM NaN_3, 0.5 mM DSS and 5% D_2O. The final

concentration in the NMR samples was 160 µM and 70 µM for CB2 and ^{C105S}CB2, respectively.
NMR measurements were performed on a Bruker NEO 500 MHz NMR spectrometer equipped with a TCI cryogenic probe head with Z gradient. ^{1}H-^{15}N HSQC spectra were acquired using a standard Bruker pulse sequence with an acquisition time of 140 ms and 256 increments, with 16 scans for CB2 and 160 scans for ^{C105S}CB2. Total measurement time was 1 h 20 min and 13 h 30 min for CB2 and ^{C105S}CB2, respectively. All NMR experiments were performed at 298 K. The spectra were processed and analyzed using Bruker Topspin 4.1.4.

Cell culture

All B cell lymphoma cell lines, except for SC-1, were a kind gift by Uta Hoepken and Armin Rehm (MDC Berlin). SC-1 cells were acquired from DSMZ. All B cell lymphoma cell lines and the Jurkat cell line were cultured in RPMI + 10% fetal calf serum + 2 mM glutamine + 1x penicillin/streptomycin (for B cell lymphoma: + 1 mM sodium pyruvate). MCF-7 cells were cultured in DMEM + 10 % FCS + 2 mM glutamine + 1x penicillin/streptomycin. MCF-10A cells were cultured in DMEM/F12 (1:1) + 5 % horse serum + 20 ng/mL EGF + 0.5 mg/mL hydrocortisone + 100ng/mL cholera toxin + 10 µg/mL insulin + 2 mM glutamine + 1x penicillin/streptomycin. All cell lines were cultured at 37 °C, 5 % CO_2. Suspension cells were diluted into fresh medium every 2-3 days. Adherent cells were passaged and diluted every 3-4 days when confluent using trypsin/EDTA. All cell lines were tested for mycoplasma contamination on a monthly basis.

Nanobody binding assays and determination of apparent affinity

For flow cytometry screenings, one million cells per sample of the respective cell line or healthy human peripheral blood mononuclear cells (PBMCs) were stained with 24 µM nanobody solution or PBS only (negative control). Secondary staining was conducted with 50 µl anti-6X His Tag ATTO 647N conjugated antibody (1:750, Rockland Immunochemicals, Inc.) or MonoRab™ Rabbit Anti-Camelid VHH Cocktail iFluor 647 (1:500, GenScript). Stained samples were measured on a FACSCanto™ II device (BD Biosciences). Events were gated for single cells and the median fluorescence intensity (MFI) of the APC-A signal was exported using the software FlowJo (V10.8.1). MFIs were

subtracted with the negative control for background correction. MFIs from three independent experiments were determined and compared to examine differences in cell binding. For the determination of apparent affinity via flow cytometry, SC-1 cells were incubated as described above with different concentrations of CB2 (0.05-22 µM). Bound fractions of cells were determined with the software FlowJo and for each concentration, the relative binding was calculated based on the maximum binding at saturation. Data from three independent experiments was plotted and fitted using a 2-parameter sigmoidal regression model with the following equation:

$$y = \frac{1}{1 + \exp\left(\frac{-x + x_0}{b}\right)}$$

Apparent affinity (K_D*) was exported from the fit by determination of x_0. Error bars of the data points as well as the error (±) of the K_D* values correspond to standard errors.

For binding studies using confocal microscopy, two million cells per sample were washed once with PBS and resuspended in 50 µL of CB2 or ^{C105S}CB2 (24 µM) in PBS. The negative control was resuspended in PBS. Cells were incubated 1 h at RT and shaking at 400 rpm. After three washes with PBS, cells were incubated with 50 µL anti-6X His Tag Atto 647N (1.3 µg/mL, Rockland Inc.) for 1 h at RT in the dark at 400 rpm. Cells were washed three times with PBS and resuspended in 1 mL PBS. They were settled on 25 mm coverslips in 6-well plates for 30 min in the dark at RT. Afterwards the supernatants were aspirated carefully and 750 µL of 4% paraformaldehyde (PFA) + 0.2% glutaraldehyde (GA) were added to each well for fixation. Samples were fixed at RT for 15 min in the dark. Afterwards, the coverslips were washed three times with 1 mL PBS, flipped onto a microscopy glass slide prepared with 50 µL of Roti®Mount FluorCare DAPI mounting solution (Carl Roth, Darmstadt, Berlin) and sealed with transparent nail polish. The slides were examined and imaged under a LSM700 laser scanning confocal microscope (Zeiss). The images were analyzed using Fiji (Fiji Is Just ImageJ) by manually selecting cells and then measuring their fluorescence intensity (mean grey value). The mean fluorescence intensity (MFI) was normalized using the following equation:

$$Normalized\ MFI\ (x) = \frac{MFI(x) - MFI\ (PBS)}{MFI(CB2) - MFI\ (PBS)}$$

The statistical analysis was performed with the software GraphPad Prism 9.3.1 (GraphPad Software, Inc.).

Thiol-binding inhibition assays

For DTT inhibition experiments, incubation of CB2 and SC-1 cells was carried out in the presence of 1 mM DTT. Anti-CD19-PE (1:100, BioLegend) was used as positive control Ab, as DTT should not affect binding of this Ab to B cells. To confirm structural integrity of the anti-CD19-PE antibody upon DTT treatment, non-reducing SDS-PAGE was performed with the antibody incubated in PBS only or 1 mM DTT for 1 h. Samples were loaded with native PAGE buffer. As a reduced control, one additional sample was treated with reducing SDS-PAGE sample buffer before loading. Antibody integrity was visualized via the fluorescence signal of PE in the gel. For tris(2-carboxyethyl)phosphine (TCEP) and reduced glutathione (GSH) inhibition assays, SC-1 cells were incubated with CB2 in the presence of 0.5 mM TCEP or 4 mM GSH, respectively.

For NEM inhibition experiments, CB2 was pre-incubated with 1.2 mM NEM for 2 h at RT. Excess NEM was washed out thrice using an Amicon Ultra Centrifugal Filter (cut-off = 10 kDa) and NEM-pre-treated CB2 was used for incubation with SC-1 cells. Nanobody binding was detected with MonoRab™ Rabbit Anti-Camelid VHH Cocktail iFluor 647 (1:500, GenScript).

Determination of surface thiol levels

Peripheral blood mononuclear cells (PBMCs) were freshly isolated as described elsewhere[2]. One million PBMCs or SC-1 cells were pelleted and incubated with AF647-maleimide (1:1,000, Jena Bioscience) or PBS for 15 min on ice in the dark[3]. Cells were washed thrice with PBS and analyzed by flow cytometry. Additional samples were prepared the same way and confocal microscopy was used to confirm that AF647-maleimide only stains the cell membrane but not intracellular compartments. The mean fluorescence intensity from three independent FACS experiments was quantified and

background-corrected. Differences between healthy lymphocytes and SC-1 cells were tested for significance using student's t-test in Origin.

Synthesis of Rha_3

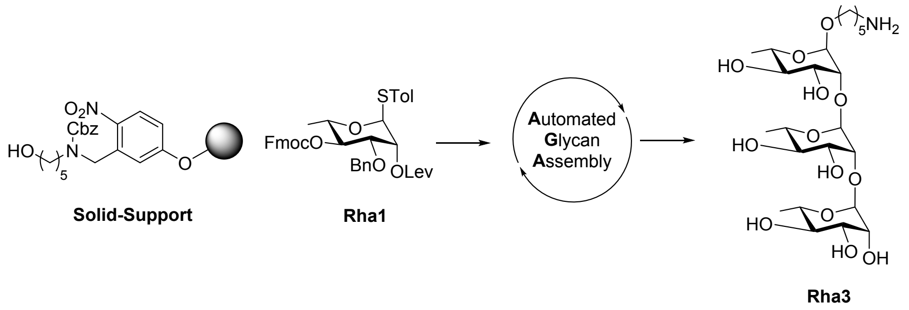

Repeat	Building Blocks	Modules	Notes
3x		**I** – Acidic Wash	
	Rha1 (2 x 5.0 equiv.)	**IIa** – Glycosylation with thioglycoside – 2 cycles	-20 °C (T_1) 10 min (t_1)<br>0 °C (T_2) 30 min (t_2)
		**III** – Capping	
		**IVc** – Lev Deprotection	

The automated synthesis of Rha_3 was performed on a home-built synthesizer developed at the Max Planck Institute of Colloids and Interfaces using modules reported earlier[4]. Protected Rha_3 (35 mg, 0.022 mmol, crude yield: 80%) was obtained as a colorless oil after photo-cleavage from solid support with a UV-150 Medium-Pressure Mercury Lamp (arc length 27.9 cm, 450 W) surrounded by a long-pass UV filter (Pyrex, 50% transmittance at 305 nm). Deprotection of Rha_3 was achieved by methanolysis and hydrogenolysis. Briefly, to a solution of protected oligosaccharide in $MeOH:CH_2Cl_2$ (2 mL, 1:1), sodium methoxide (0.5 M solution in MeOH, 2.2 equiv. per ester group) was added. The mixture was stirred at room temperature for 2 h. Then Amberlite IR-120 (H^+ form) was added to quench. After neutralization, the reaction mixture was filtered and the solvent was removed in vacuo. The crude compound was dissolved in 4 mL of EtOAc:t-BuOH:H_2O (2:1:1). Pd/C (10%) was added to the solution and the suspension was stirred in a H_2 bomb with 60 psi pressure over night. The insoluble material was removed by a CHROMAFIL ®Xtra, RC 0.45 syringe filter. The solid was washed once with t-BuOH and several times with water. The filtrate was collected and concentrated in vacuo. Crude

products were dissolved in water and analyzed/purified using analytical/preparative HPLC. A Thermo-Scientific Hypercarb column (150 mm x 4.60 mm I.D.) was used for analytical RP-HPLC with a flow rate of 0.70 mL/min with water (0.1% HCO_2H)/acetonitrile as eluents (100% H_2O (0.1% HCO_2H) for 5 min, 0 → 30% acetonitrile in H_2O (0.1% HCO_2H) over 30 min, 30 → 100% acetonitrile in H_2O (0.1% HCO_2H) over 5 min, 100% acetonitrile for 5 min). This yielded deprotected, pure Rha_3 (6 mg, 0.011 mmol, 41%) as a white solid after lyophilization. NMR spectra confirming the purity of the compound can be found in Fig. S4.

^{1}H NMR (700 MHz, D_2O): δ 5.11 (s, 1H), 4.99 (s, 1H), 4.87 (s, 1H), 4.09 (ddd, J = 11.5, 3.4, 1.7 Hz, 2H), 3.95 – 3.83 (m, 3H), 3.82 – 3.67 (m, 5H), 3.56 (dt, J = 10.0, 6.1 Hz, 1H), 3.53 – 3.43 (m, 3H), 3.01 (t, J = 7.6 Hz, 2H), 1.76 – 1.61 (m, 4H), 1.52 – 1.41 (m, 2H), 1.31 (d, J = 6.3 Hz, 3H), 1.30 (d, J = 6.2 Hz, 3H), 1.28 (d, J = 6.2 Hz, 3H) ppm. **^{13}C NMR** (176 MHz, D_2O): δ 102.2, 100.9, 98.4, 78.5, 78.3, 72.1, 72.1, 72.0, 70.2, 70.1, 70.0, 69.8, 69.3, 69.2, 68.8, 67.7, 39.4, 28.0, 26.6, 22.5, 16.7, 16.6, 16.6 ppm. **HRMS** (QToF): Calcd for $C_{23}H_{44}NO_{13}$ $[M + H]^+$ 542.2807; found 542.2813.

Rha_3 conjugation to CB2

Rha_3 was coupled to CB2 in a two-step reaction to achieve multivalent display while maintaining CB2 binding activity. First, Rha_3 was coupled to the synthetic peptide thFF03 (sequence: GGGLKKELAALKKELAALKK, synthesized by ProteoGenix). The peptide thFF03 is a truncated, glycylated version of the previously published hFF03[5] and was chosen because its many lysines allow chemical conjugation, while the N-terminal glycines make it amenable to srtA conjugation. Rha_3 with an aminopentanol linker was conjugated to thFF03 as described elsewhere[6]. Briefly, 1 eq. of Rha_3 was mixed with 10 eq. of homobifunctional adipic acid *p*-nitrophenyl diester in 300 µL DMSO + 25 µL pyridine + 10 µL triethylamine and stirred for 3 h at 300 rpm, RT. After lyophilization, esterified Rha_3 was washed 3x with a 1:1 mixture of diethyl ether and dichloromethane and 3x with a 1:4 mixture of the same solvents until uncoupled linker was no longer detected in the wash fractions by UV light. The absence of glycan in the wash fractions was confirmed by thin-layer chromatography. Then, 6 eq. of esterified Rha_3 was mixed with 1 eq. of thFF03 in 140 µL conjugation buffer (0.1 M sodium phosphate pH 8) and stirred for 24 h at 70 rpm, RT. Successful conjugation and degree of loading was assessed by MALDI and the glycopeptide (hereon Rha-thFF03) was purified by HPLC on a C18 column.

In a second step, Rha-thFF03 was conjugated to CB2 via srtA coupling. 15 nmol of CB2, 3.75 nmol of srtA, 406 nmol of Rha-thFF03 and 40 µL of equilibrated HisPur Ni-NTA resin (ThermoFisher) were mixed in 300 µL of srtA buffer (150 mM NaCl, 10 mM CaCl2, pH 7.5) and incubated rotating for 30 min at 4 °C. Resin was pelleted (700 g, 2 min) and the supernatant was checked for the presence of CB2-Rha-thFF03 by SDS-PAGE and MALDI and brought into PBS via Amicon® Ultra centrifugal filter units (MWCO = 10 kDa).

Glycan array

Human IgG/IgM Abs were purified from human serum (Seraclot, PanBiotech) via protein A/G column (ThermoFisher). The glycans were dissolved at 0.1 mM in 50 mM sodium phosphate buffer pH 8.5 and printed in 64 identical fields to NHS activated hydrogel glass slides (CodeLink slides, Surmodics) using a non-contact S3 microarray spotter (Scienion, Berlin, Germany). After incubation overnight in a humidified box, the remaining NHS groups of the slides were quenched with ethanolamine. The slides were blocked with 1% (w/v) bovine serum albumin (BSA) in phosphate buffered saline (PBS) and a 64 well incubation gasket (FlexWell Grid, Grace Bio Labs) was attached. The slides were incubated with human serum (Sigma-Aldrich, Darmstadt, Germany) or purified Abs diluted 1:100 in 1% BSA-PBS for 1 h at 37°. After three washes with PBS containing 0.1% (v/v) Tween-20 (PBS-T) the slides were incubated with goat anti-human IgG (H+L) AlexaFluor 647 (Invitrogen, Cat. A21445) and goat anti-human IgM (µ chain) AlexaFluor 488 (Invitrogen, Cat. A21215) diluted 1:400 for 1 h at 37°C. The slides were washed twice with PBS-T. After removing the gasket, the slides were washed once with PBS and once with water. The dried slides were scanned with a GenePix 4300A microarray scanner (Molecular Devices). Intensities were evaluated with GenePix Pro 7 (Molecular Devices). The statistical analysis was performed with the software GraphPad Prism 9.3.1 (GraphPad Software, Inc.).

Complement activation assay

Human IgG/IgM Abs (0.2 mg/mL) were mixed with CB2 or CB2-Rha-thFF03 (0.4 mg/mL) and incubated for 1 h at RT. Two aliquots of one million SC-1 cells each were incubated with the CB2 or CB2-Rha-thFF03 Ab mixtures for 1 h at RT, 400 rpm. Two samples

incubated with goat anti-human IgG Ab (0.24 mg/mL, Invitrogen) were included as positive controls because this Ab binds to the surface IgGs of B cell lymphoma. After 1 h incubation, cells were pelleted, resuspended in 50 µL of active or heat-inactivated (30 min at 56 °C) rabbit complement (Cedarlane) and incubated for 2 h at 37 °C. Cells were again pelleted, resuspended in 200 µL of 7-AAD (1:100, BioLegend) and cell viability was measured on a FACSCanto II (BD Bioscience).

Cy3 conjugation to CB2

Cy3 conjugates were prepared as described previously[7]. 100 µM CB2 or ^{C105S}CB2, 25 µM srtA and 3 mM dibenzocyclooctyne-NH2 (DBCO-amine) were mixed in 1.2 mL srtA buffer (150 mM NaCl, 10 mM CaCl2, pH 7.5) for 30 min at 4° C with 180 µL Ni-NTA resin on a rotation wheel for srtA-mediated functionalization of sortagged nanobodies with dibenzocyclooctyne-NH2 (DBCO-amine). The supernatant containing CB2-DBCO or ^{C105S}CB2-DBCO was collected, and remaining DBCO-amine was removed using an PD MidiTrap™ G-10 column (Cytiva, Freiburg, Germany). Nanobody-DBCO conjugate was concentrated in Amicon® Ultra centrifugal filter units (MWCO = 3 kDa) at 4° C, 3,200 g and concentrations were determined by NanoDrop® ND-1000. The successful conjugation and purity were analyzed on an SDS-Page and the conjugate was stored at 4° C until proceeding with the click-reaction.

40 µM CB2-DBCO or ^{C105S}CB2-DBCO conjugate was mixed with 80 µM Cy3-azide (Jena Bioscience) in click reaction buffer (20 mM HEPES pH 7.5, 300 mM NaCl and 10% glycerol) and incubated for 17 h. Unbound reaction partners were removed by using an PD MidiTrap™ G-10 column (Cytiva, Freiburg, Germany). Successful labeling was verified by reducing SDS-PAGE and FACS measurement. The conjugate (CB2-Cy3 or ^{C105S}CB2-Cy3) was stored at 4°C and used for internalization assays within a week.

Endocytosis inhibition assay

For endocytosis inhibition assays, 1.5 million SC-1 cells were pre-treated for 30 min with 80 µM dynasore or chlorpromazine. Non-treated cells were included as positive control. Cells were then incubated with 100 µL of CB2-Cy3 or ^{C105S}CB2-Cy3 (0.5 mg/mL) for 2 h at RT, 400 rpm in the dark in the presence of the respective inhibitor or PBS. Cells were

pelleted, washed once with 1 mL ice-cold acidic wash buffer (100 mM glycine, 150 mM NaCl, pH 2.2), to remove surface-bound protein, and twice with PBS. Cell pellets were resuspended in 250 µL each and settled onto 12 mm coverslips for 30 min at RT in the dark. The suspension was carefully aspirated, and cells were fixed with 250 µL fixative (4% paraformaldehyde + 0.2% glutaraldehyde in PBS) for 15 min at RT in the dark. Coverslips were washed thrice with PBS, mounted onto a drop of Fluoromount G (ThermoFisher), sealed and stored in the dark till imaging.

Live-cell intracellular traffic assay

For live-cell imaging of CB2 internalization, 2 million SC-1 cells were washed twice in PBS by pelleting for 5 min at 300 g and resuspended in RPMI 1640 medium supplemented with 10% FCS, 2 mM glutamine, 1 mM sodium pyruvate, 100 mM HEPES. Next, cells were incubated for 30 min at RT with 1 µg/ml LysoTracker™ Deep Red while shaking. Cells were then washed in PBS by pelleting and plated on a 18-mm glass coverslip in RPMI 1640 medium supplemented with 10% FCS, 2 mM glutamine, 1 mM sodium pyruvate and 100 mM HEPES. Cells were allowed to settle down for 15 min at RT before adding 100 µg of either CB2-Cy3 or ^{C105S}CB2-Cy3. Cells were then imaged live at the indicated time points after protein addition on a spinning disk confocal microscope consisting of an inverted Olympus IX71 microscope equipped with a Yokogawa CSU-X1 spinning disk, a 60×/1.42 NA oil Olympus objective and an sCMOS camera (Hamamatsu). Z-stacks were acquired during measurement to ensure imaging of the whole organelles.

Colocalization analysis pipeline

The % of colocalization between the two channels imaged (nanobody-Cy3 and LysoTracker™ Deep Red) was quantitatively determined on a per-object basis using a custom-made pipeline in CellProfiler[8]. In brief, the Z-stacks acquired for each channel were first split into individual images that were then segmented into objects. The % of colocalization was calculated as the amount of overlapping pixels between the identified objects in the two channels, divided by the total pixel area occupied by the CB2-Cy3/^{C105S}CB2-Cy3 channel. The values obtained for the three individual experiments

were pooled together, and a one-way ANOVA with Tukey's *post-hoc* analysis was performed in Origin(Pro) Version 2020 (OriginLab) to test for significant differences.

MMAE conjugation to CB2

MMAE was conjugated to CB2 via srtA coupling. 30 nmol of CB2, 7.5 nmol of srtA, 900 nmol of Gly_3-Val-Cit-PAB-MMAE (MedChemExpress, in DMSO) and 80 µL of equilibrated HisPur Ni-NTA resin (ThermoFisher) were mixed in 600 µL of srtA buffer (150 mM NaCl, 10 mM CaCl2, pH 7.5) and incubated rotating for 30 min at 4 °C. Resin was pelleted (700 g, 2 min) and the supernatant was checked for the presence of CB2-MMAE by SDS-PAGE and MALDI. The supernatant was dialysed with 3x 2 L PBS to remove any uncoupled drug and concentrated via Amicon® Ultra centrifugal filter units (MWCO = 10 kDa) before estimating the protein concentration via microBCA kit (ThermoFisher). Drug conjugates were filtered through 0.22 µm and stored at 4°C.

Drug delivery assay

In a 96-well plate, triplicates of CB2-MMAE, ^{C105S}CB2-MMAE and MMAE were prepared with 10 µL at 190 nM each. Wells with unconjugated CB2 and 0.1% Triton-X100 were included as negative and positive controls, respectively. SC-1 cells were resuspended in complete medium without phenol red, and 30,000 cells in 180 µL medium were added to each well. As another control, three empty wells were filled with medium without cells. The plate was incubated for 48 h at 37°C, 5% CO_2. Next, 10 µL of WST-8 reagent (from CCK8 kit, Abcam) were added to each well and, after another incubation for 3 h at 37°C, 5% CO_2, the absorbance of the plate was measured at 460 nm with a Tecan Infinite plate reader.

Supplementary Figures

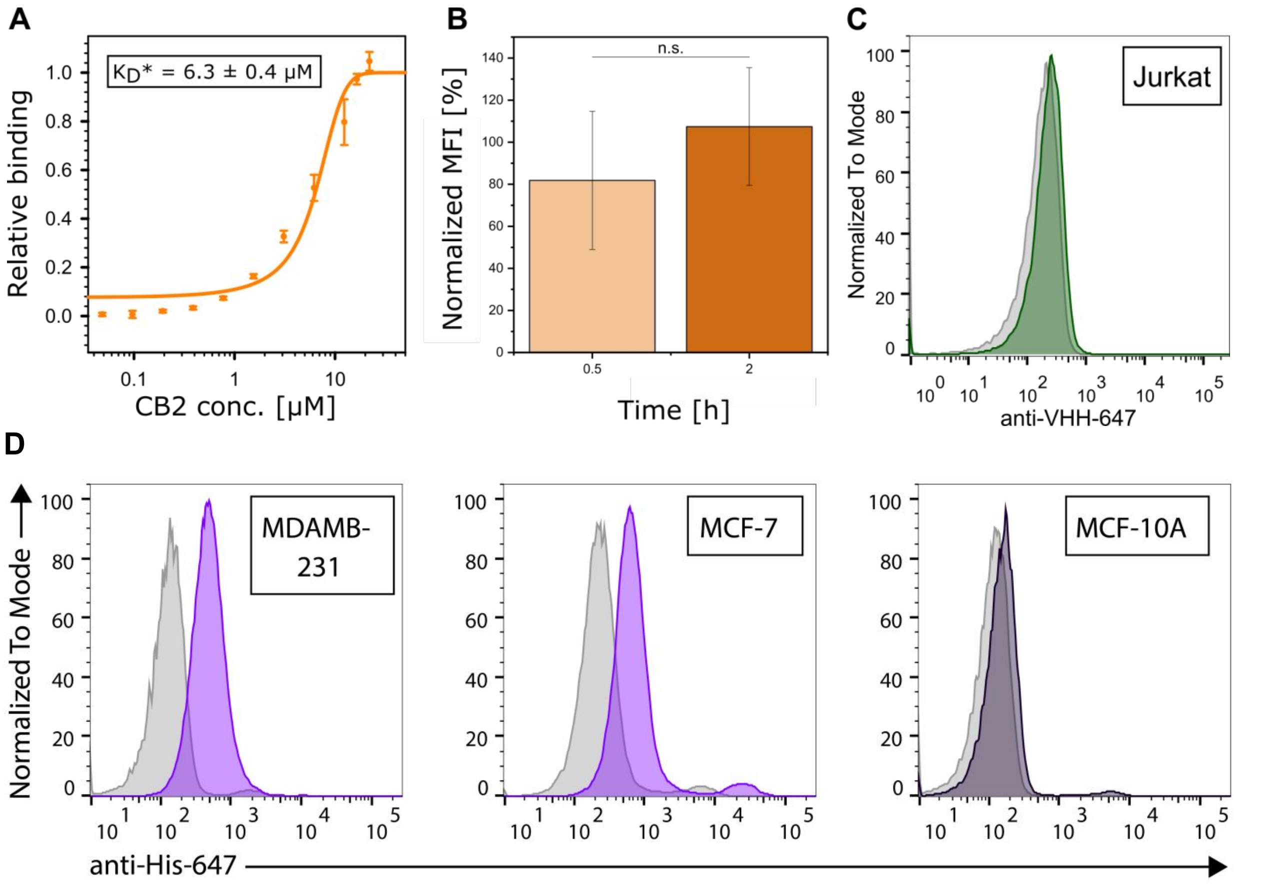

Figure S1: (A) Apparent affinity of CB2 to SC-1 cells. On-cell affinity was determined via flow cytometry incubating the cells with different concentrations of CB2 for 60 min. Data from three independent experiments is shown. Error bars of the data points and error (±) of the apparent dissociation constant (K_D*) correspond to standard errors. **(B) Time course of CB2 binding to SC-1 cells**. Binding was tested via flow cytometry after different time points (0.5 h, 1 h, 2 h) of incubation with 24 µM CB2 to show that CB2 is still partially accessible on the cell surface after prolonged incubation. MFIs were normalized to the MFI of the 1 h time point (standard assay condition). There was no significant change in binding, indicating a similar surface retention of CB2 after prolonged incubation. Data from three individual experiments is shown. Error bars represent standard errors. Significance was tested via t-test. **(C) Binding of CB2 to T cell leukemia.** CB2 does not recognize T cell leukemia cell line Jurkat. Green: CB2, grey: PBS. Y axis: normalized cell count, X axis: mean fluorescence intensity of anti-VHH 647 detection Ab. **(D) Binding of CB2 to breast**

cancer. The flow cytometry histograms show that CB2 recognizes breast cancer cell lines MCF-7 and MDAMB-231 but does not bind to non-tumorigenic breast cells MCF-10A. Purple: CB2, grey: PBS. Y axis: normalized cell count, X axis: mean fluorescence intensity of anti-His 647 detection Ab. For (C-D), cells were incubated with 24 µM CB2 for 60 min.

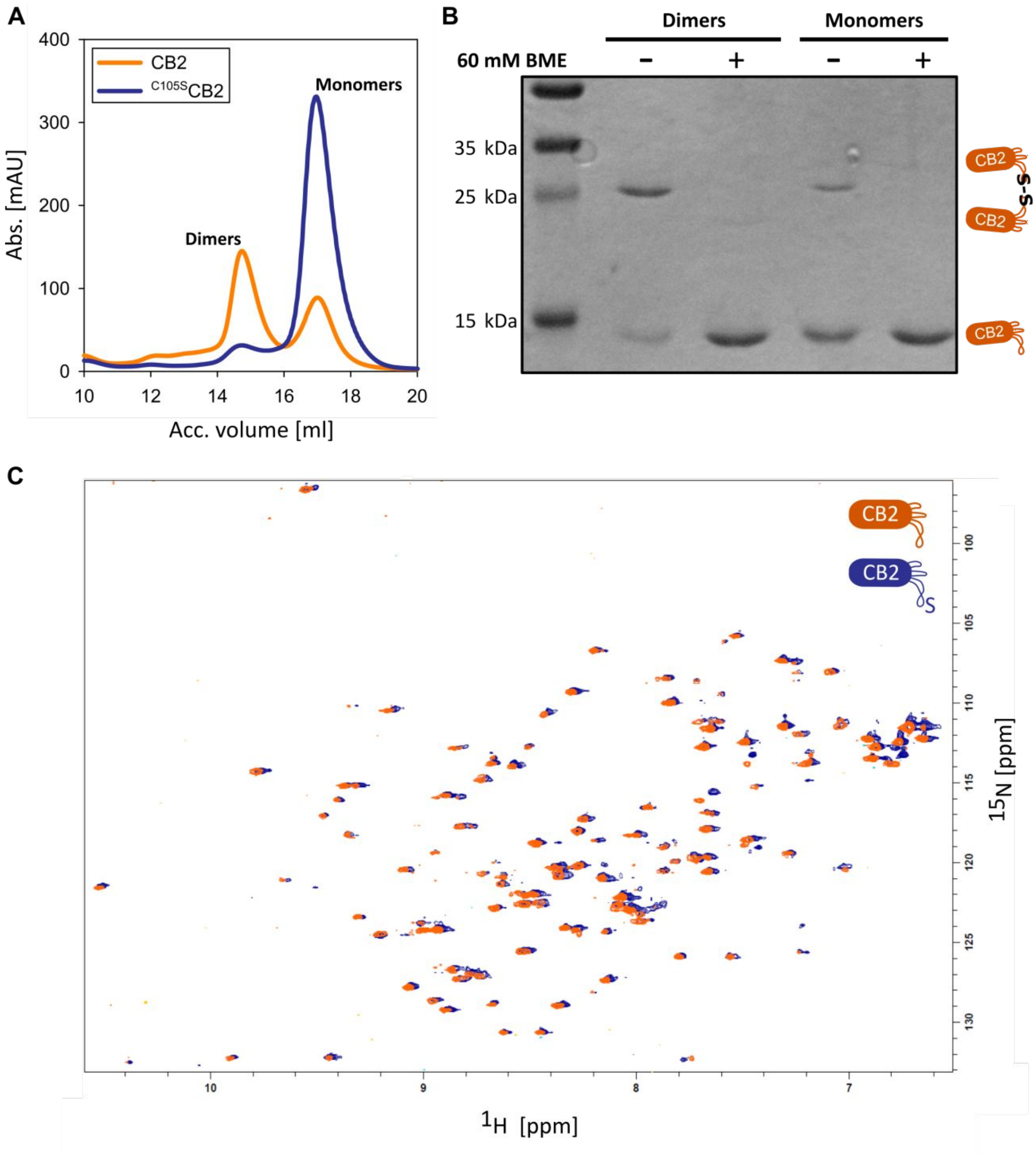

Figure S2: Characterization of CB2 and ^{C105S}CB2. (A) Comparative size exclusion chromatography (SEC) of CB2 and ^{C105S}CB2. Both proteins were purified as described in the method section but without DTT present. Subsequently, fractions were run on a

Superdex™ 200 Increase FPLC column (23 ml) to compare elution profiles. Notably, a fraction of CB2 elutes earlier than the expected volume for a nanobody monomer (≈ 17 ml), indicating the presence of multimeric complexes. This additional peak is abolished in case of ^{C105S}CB2. (B) Non-reductive SDS-PAGE of CB2 SEC fractions indicated in (A). Samples were incubated with native PAGE sample buffer with or without 60 mM beta-mercaptoethanol for 1 hour at 37°C and run on a standard SDS-PAGE gel. The gel shows that the additional SEC peak of CB2 represents dimers that are completely converted into monomers upon exposure to beta-mercaptoethanol. Replacement of cysteine-105 by serine prevents the dimerization. (C) Overlaid ^{1}H–^{15}N HSQC spectra of CB2 (orange) and ^{C105S}CB2 (blue). No major signal shifts are visible, indicating the non-disruptive nature of the C105S mutation. CB2 and ^{C105S}CB2 were acquired at 160 µM and 70 µM, respectively, in PBS buffer supplied with 0.75 mM DTT, 8 mM NaN_3, 0.5 mM DSS and 5% D_2O at 298 K.

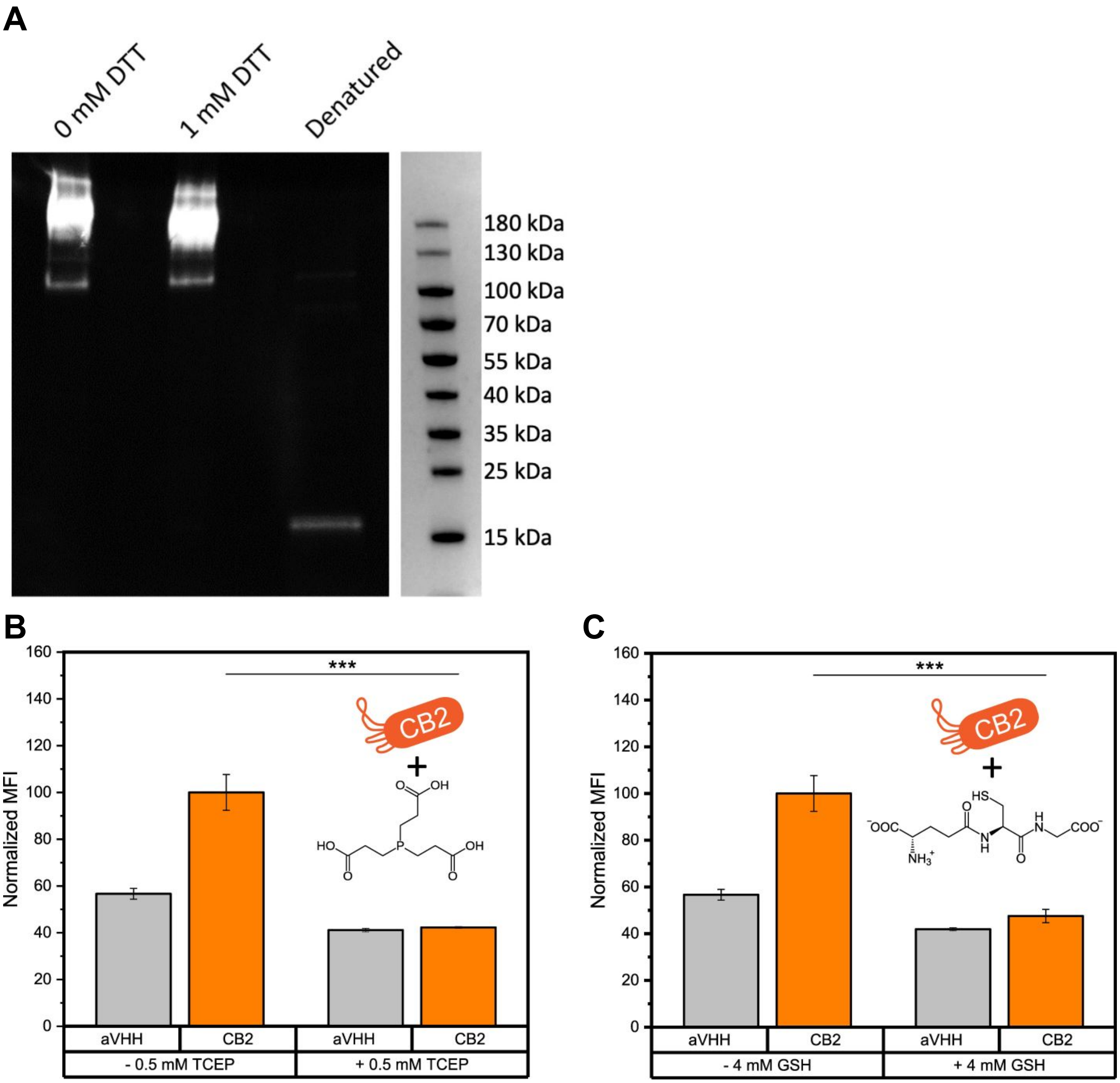

Figure S3: (A) Small amounts of DTT do not reduce the pre-existing disulfide bonds of the anti-CD19-PE Ab. SDS-PAGE of anti-CD19-PE in the absence and presence of 1 mM DTT. The Ab was detected via fluorescence of the conjugated PE. As denatured control, the Ab was boiled with SDS-PAGE sample buffer containing 100 mM DTT. **(B-C) CB2 binding is abolished in the presence of TCEP (B) and GSH (C).** Values represent mean fluorescence intensity (MFI) from n = 3. Error bars represent the standard error of the mean (SEM). Differences were tested for significance using one-way ANOVA followed by Tukey's *post-hoc* test with (***) $p < 0.001$. Cells were incubated with 24 µM CB2 in the presence of the respective reducing agent for 60 min.

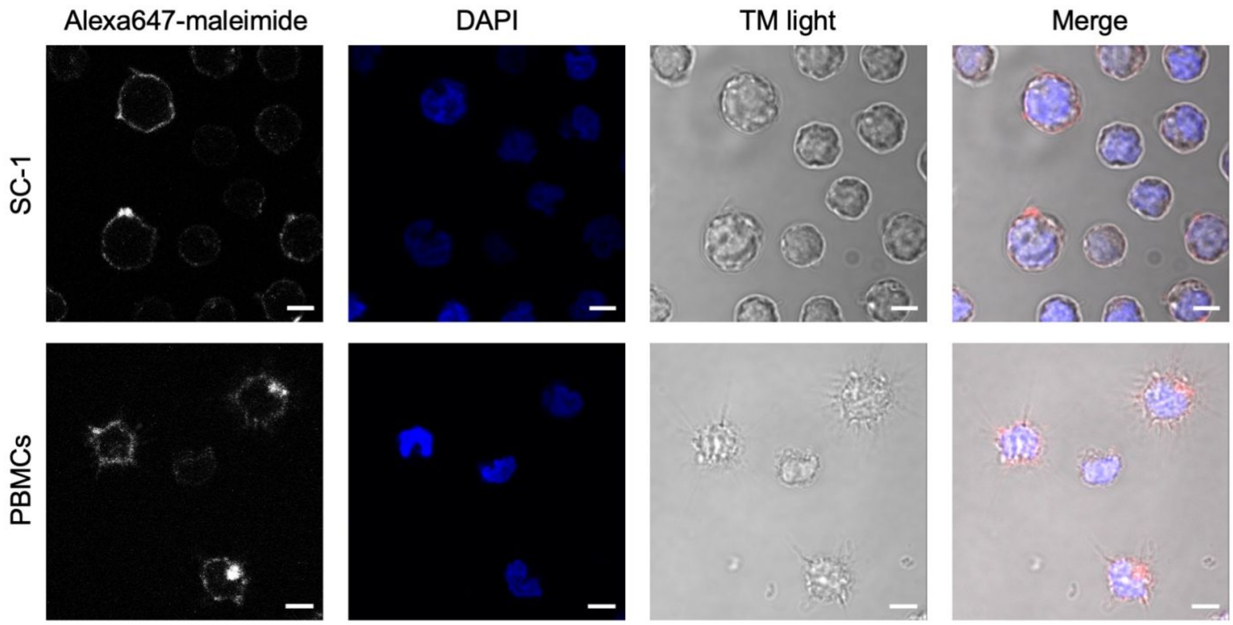

Figure S4: Alexa647-maleimide stains cell surface thiols. Confocal microscopy confirms that Alexa647-maleimide signal is only detected on the cell surface but not inside the cell. Upper panel: SC-1 lymphoma, lower panel: PBMCs from healthy human donors. Colors in merge: red = Alexa647-maleimide, blue = DAPI. Scale bar = 5 µm.

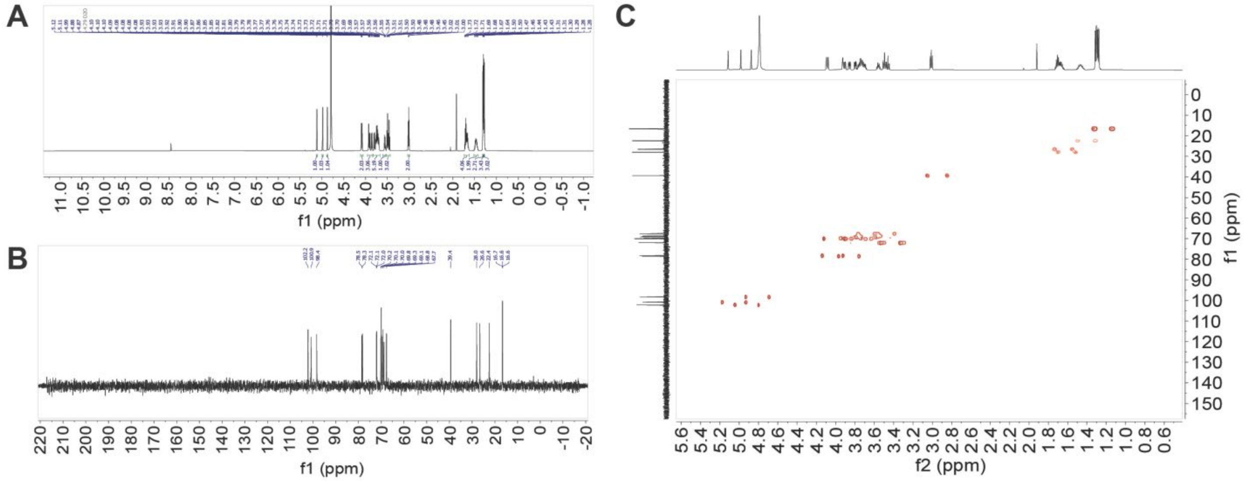

Figure S5: NMR spectra of Rha₃. (A) ^{1}H NMR (700 MHz, D_2O). (B) ^{13}C NMR (176 MHz, D_2O). (C) Coupled ^{13}C,^{1}H HSQC.

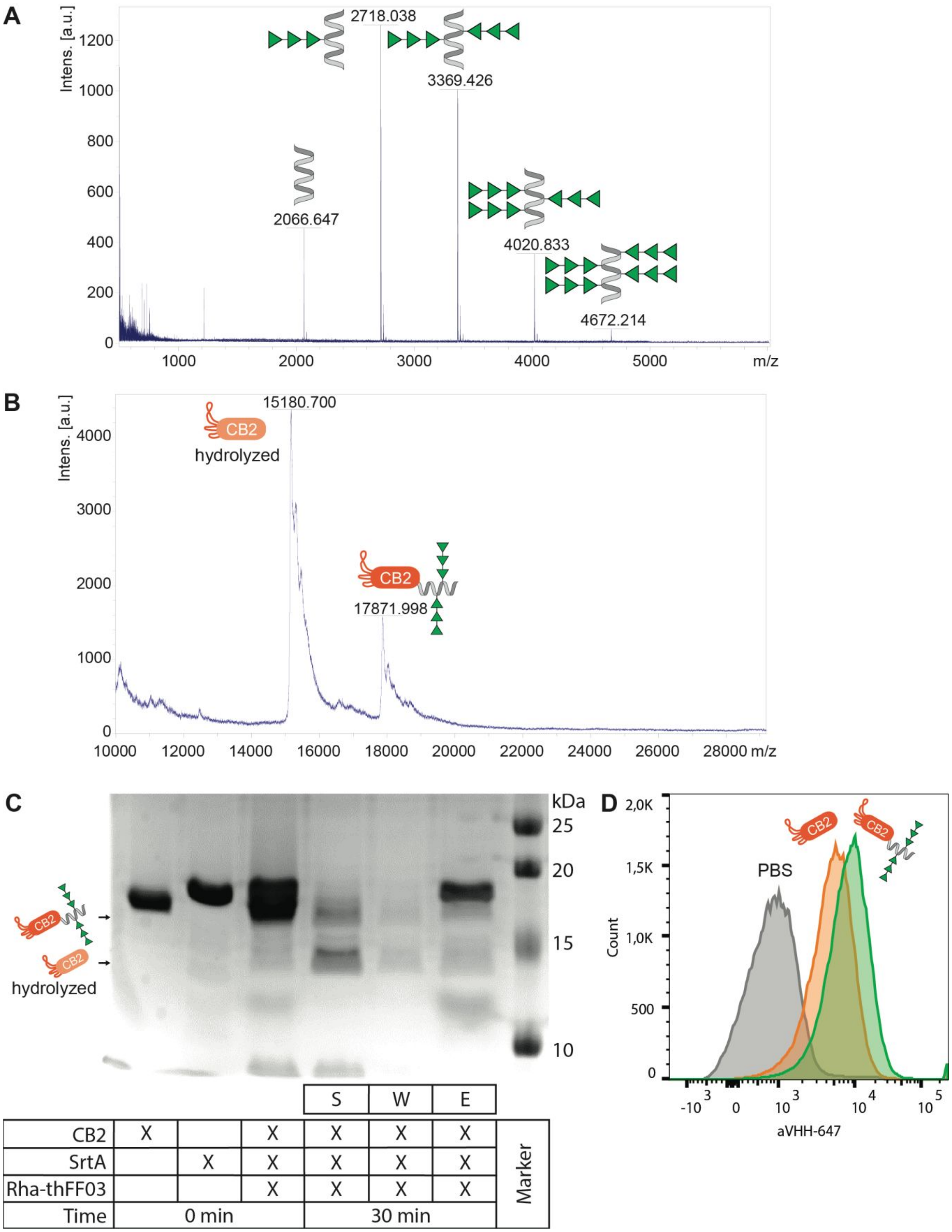

Figure S6: Conjugation of Rha-thFF03 to CB2. (A) Chemical conjugation of Rha_3 to thFF03. The MALDI spectrum shows that 0-4 molecules of Rha_3 were deposited per peptide. (B-C) SrtA-mediated conjugation of Rha-thFF03 to CB2. (B) MALDI spectrum

confirming the presence of CB2-Rha-thFF03 after the srtA reaction. (C) Coomassie-stained SDS-PAGE following the course of the srtA reaction over time. S, W and E correspond to supernatant, wash and eluate fractions of Ni-NTA affinity chromatography after 30 min of srtA reaction. Note that both the desired product CB2-Rha-thFF03 (as expected) but also the hydrolyzed CB2 side product are found in the supernatant. (D) Flow cytometry histogram validating that CB2-Rha-thFF03 retains binding to SC-1 cells. Cells were incubated with 24 µM CB2 or CB2-Rha-thFF03 for 60 min.

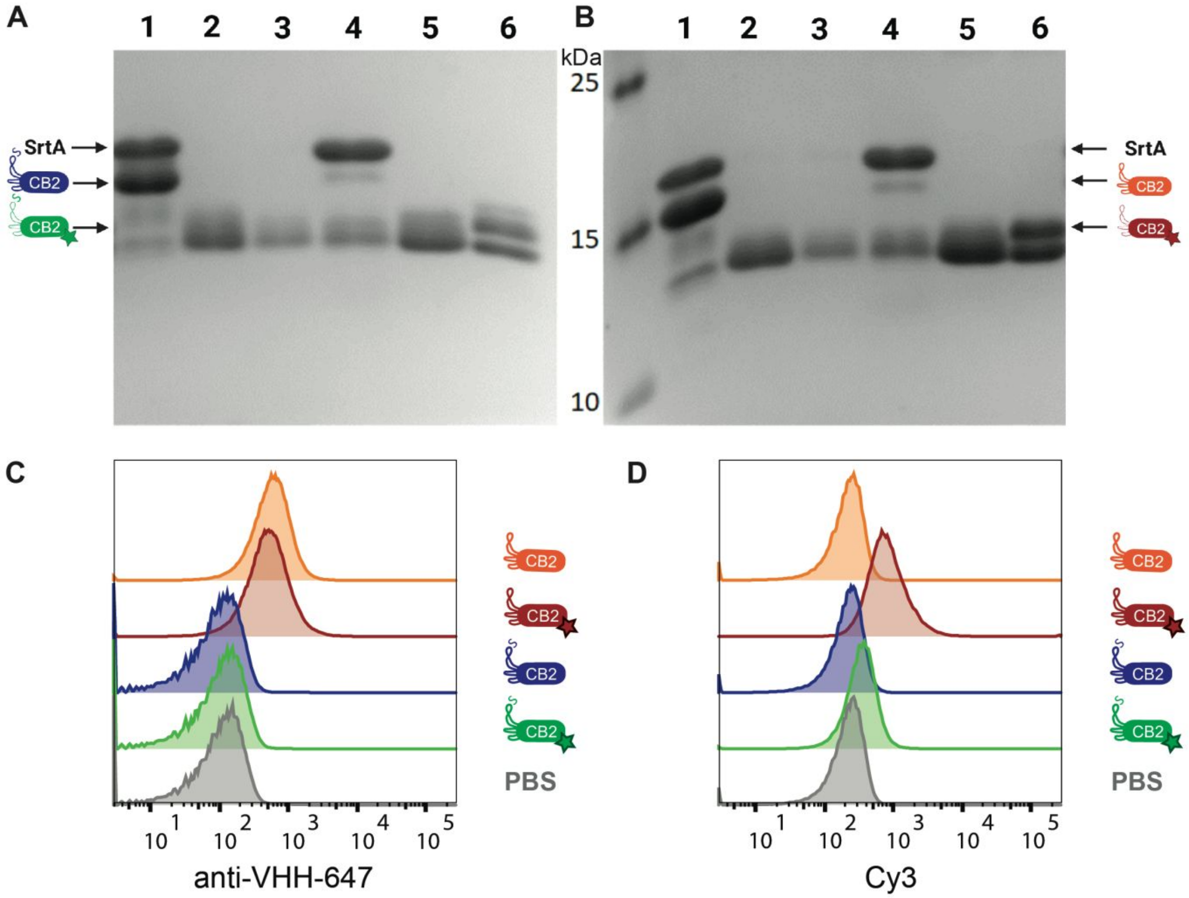

Figure S7: Conjugation of Cy3 to CB2. (A-B) Coomassie-stained SDS-PAGE gels of samples taken during the conjugation of Cy3 to (A) ^{C105S}CB2 and (B) CB2. 1 = reaction mixture after 0 min; 2 = Ni-NTA flow-through containing DBCO-modified Nb; 3 = wash of Ni-NTA beads with srtA buffer; 4 = elution of remaining His-tagged Nb and srtA; 5 = pooled and concentrated DBCO-modified Nb after PD MidiTrap^{TM} G-10 column; 6 = Nanobody Cy3 conjugate. (C-D) Binding to SC-1 cells in flow cytometry after 1 h incubation with 24 µM unconjugated and Cy3-conjugated ^{C105S}CB2, CB2 and PBS (negative control). No binding was detected for ^{C105S}CB2 and ^{C105S}CB2-Cy3.

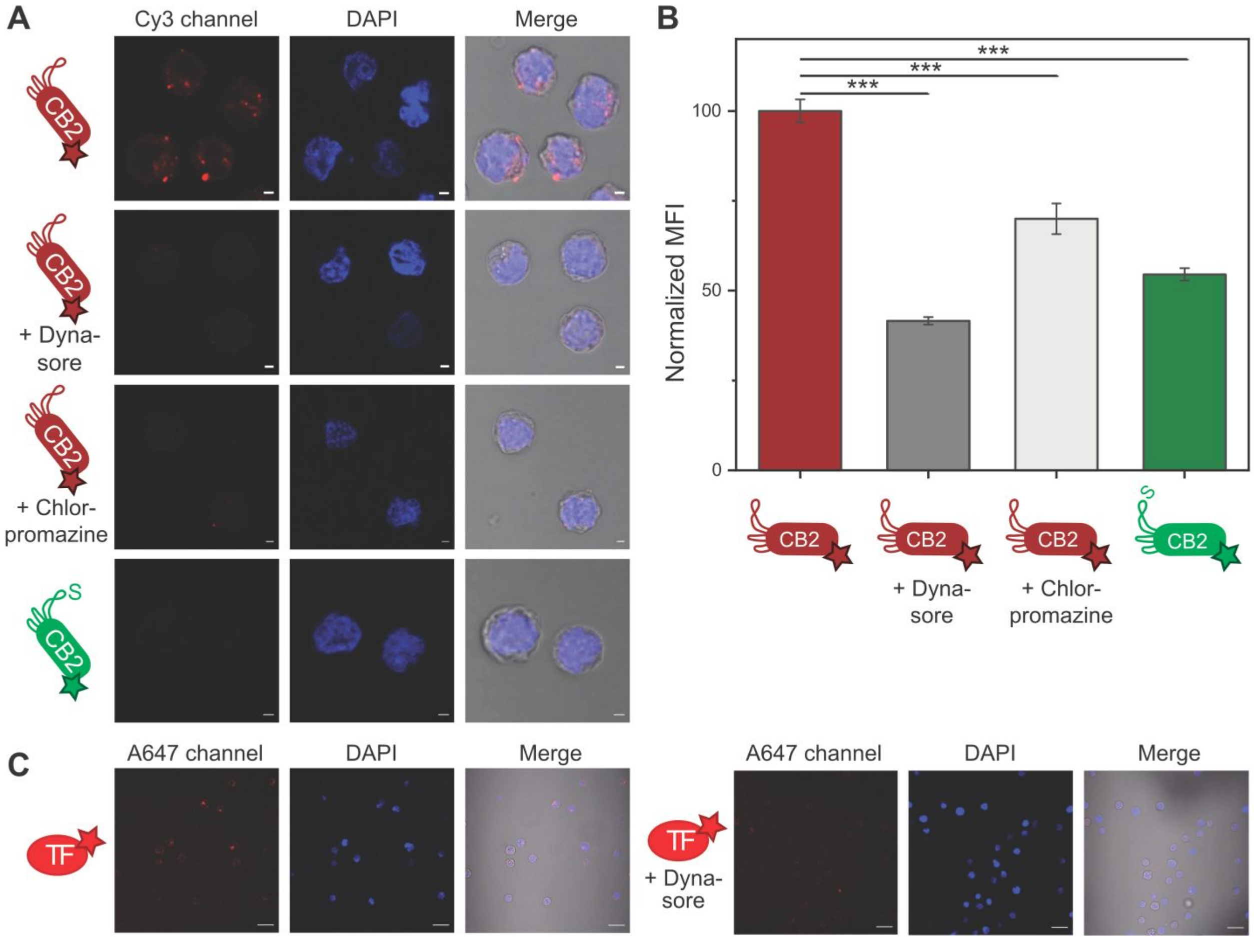

Figure S8: CB2-Cy3 internalization is affected by inhibitors of clathrin-mediated endocytosis. (A) Exemplary images of CB2-Cy3 or ^{C105S}CB2-Cy3 internalization into SC-1 cells in the presence of PBS or different inhibitors of clathrin-mediated endocytosis. Red: Cy3, blue: DAPI. Scale bar = 2 µm. (B) Quantified Cy3 fluorescence of SC-1 cells after 2 h of incubation with 28 µM CB2-Cy3, CB2-Cy3 + dynasore, CB2-Cy3 + chlorpromazine, and ^{C105S}CB2-Cy3. Note that both dynasore and chlorpromazine inhibit CB2 uptake, and that no internalization is observed for the C105S mutant. Values represent mean ± SEM (N = 3 and n > 160 cells), (***) $p < 0.001$. (C) Transferrin (TF) was used as a positive control for clathrin-mediated endocytosis. TF-Alexa647 uptake in the absence (left) or presence (right) of dynasore is detected in the red channel. Scale bar = 10 µm.

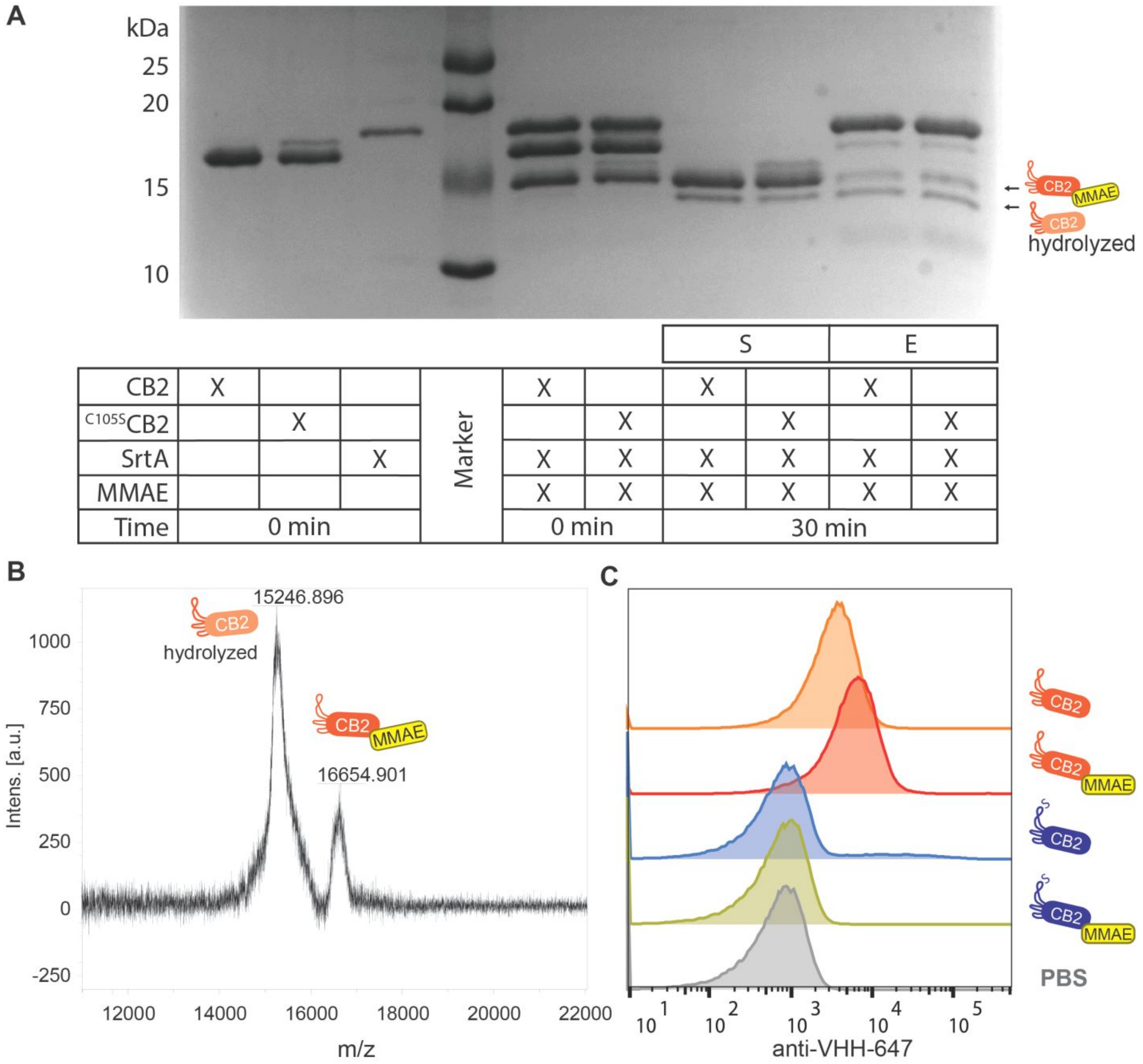

Figure S9: Conjugation of MMAE to CB2. (A) Coomassie-stained SDS-PAGE following the course of the srtA reaction over time. S and E correspond to supernatant and eluate fractions when after 30 min of srtA reaction the conjugate is purified via Ni-NTA affinity chromatography. The band intensity in the supernatant was quantified to determine the ratio of CB2-MMAE to hydrolyzed side product. (B) MALDI spectrum of the supernatant fraction from (A) confirming the presence of CB2-MMAE but also the hydrolyzed side product. (C) Flow cytometry histograms validating that CB2-MMAE retains full binding activity during conjugation. Cells were incubated with nanobody conjugates (24 µM) for 60 min. As expected, ^{C105S}CB2-MMAE does not bind to SC-1 cells.

References

(1) Heckman, K. L.; Pease, L. R. Gene Splicing and Mutagenesis by PCR-Driven Overlap Extension. *Nat. Protoc.* **2007**, *2* (4), 924–932. https://doi.org/10.1038/nprot.2007.132.

(2) Fuss, I. J.; Kanof, M. E.; Smith, P. D.; Zola, H. Isolation of Whole Mononuclear Cells from Peripheral Blood and Cord Blood. *Curr. Protoc. Immunol.* **2009**, *85*. https://doi.org/10.1002/0471142735.im0701s85.

(3) Sahaf, B.; Heydari, K.; Herzenberg, L. A.; Herzenberg, L. A. Lymphocyte Surface Thiol Levels. *Proc. Natl. Acad. Sci.* **2003**, *100* (7). https://doi.org/10.1073/pnas.2628032100.

(4) Danglad-Flores, J.; Leichnitz, S.; Sletten, E. T.; Abragam Joseph, A.; Bienert, K.; Le Mai Hoang, K.; Seeberger, P. H. Microwave-Assisted Automated Glycan Assembly. *J. Am. Chem. Soc.* **2021**, *143* (23). https://doi.org/10.1021/jacs.1c03851.

(5) Hellmund, K. S.; von Lospichl, B.; Böttcher, C.; Ludwig, K.; Keiderling, U.; Noirez, L.; Weiß, A.; Mikolajczak, D. J.; Gradzielski, M.; Koksch, B. Functionalized Peptide Hydrogels as Tunable Extracellular Matrix Mimics for Biological Applications. *Pept. Sci.* **2021**, *113* (2), 1–13. https://doi.org/10.1002/pep2.24201.

(6) Wu, X.; Ling, C.-C.; Bundle, D. R. A New Homobifunctional *p* -Nitro Phenyl Ester Coupling Reagent for the Preparation of Neoglycoproteins. *Org. Lett.* **2004**, *6* (24), 4407–4410. https://doi.org/10.1021/ol048614m.

(7) Fabricius, V.; Lefèbre, J.; Geertsema, H.; Marino, S. F.; Ewers, H. Rapid and Efficient C-Terminal Labeling of Nanobodies for DNA-PAINT. *J. Phys. D. Appl. Phys.* **2018**, *51* (47), 474005. https://doi.org/10.1088/1361-6463/AAE0E2.

(8) Carpenter, A. E.; Jones, T. R.; Lamprecht, M. R.; Clarke, C.; Kang, I. H.; Friman, O.; Guertin, D. A.; Chang, J. H.; Lindquist, R. A.; Moffat, J.; Golland, P.; Sabatini, D. M. CellProfiler: Image Analysis Software for Identifying and Quantifying Cell Phenotypes. *Genome Biol.* **2006**, *7* (10). https://doi.org/10.1186/gb-2006-7-10-r100.
